# Transcription Regulator YgeK Affects the Virulence of Avian Pathogenic *Escherichia coli*

**DOI:** 10.3390/ani11113018

**Published:** 2021-10-20

**Authors:** Jian Tu, Dandan Fu, Yi Gu, Ying Shao, Xiangjun Song, Mei Xue, Kezong Qi

**Affiliations:** 1 Anhui Province Key Laboratory of Veterinary Pathobiology and Disease Control, College of Animal Science and Technology, Anhui Agricultural University, Hefei 230036, China; tujian1980@126.com (J.T.); fudandan2020@126.com (D.F.); linlin296892447@163.com (Y.G.); julieshao1005@163.com (Y.S.); 13275769789@126.com (X.S.); 2 College of Animal Science and Food Engineering, Jinling Institute Technology, Nanjing 211169, China

**Keywords:** avian pathogenic *Escherichia coli*, *Escherichia coli* type III secretion system 2, YgeK, virulence, regulation

## Abstract

**Simple Summary:**

Avian pathogenic *Escherichia coli* (APEC) is the responsible pathogen for colibacillosis in poultry. Transcriptional regulator YgeK was a transcriptional regulator locating at *E. coli* type three secretion system 2 (ETT2) in APEC. However, the role of YgeK in APEC has not been reported. In this study, we found that the inactivation of YgeK in APEC decreased the flagellar formation ability, bacterial motility ability, serum sensitivity, adhesion ability, and virulence. Results suggested that the transcriptional regulator YgeK plays a crucial role in APEC virulence.

**Abstract:**

Avian pathogenic *Escherichia coli* (APEC) is the responsible pathogen for colibacillosis in poultry, and is a potential gene source for human extraintestinal pathogenic *Escherichia coli*. *Escherichia coli* type III secretion system 2 (ETT2) is widely distributed in human and animal ExPEC isolates, and is crucial for the virulence of ExPEC. Transcriptional regulator YgeK, located in the ETT2 gene cluster, was identified as an important regulator of gene expression in enterohemorrhagic *E. coli* (EHEC). However, the role of YgeK in APEC has not been reported. In this study, we performed amino acid alignment analysis of YgeK among different *E. coli* strains and generated ygeK mutant strain AE81Δ*ygeK* from clinical APEC strain AE81. Flagellar formation, bacterial motility, serum sensitivity, adhesion, and virulence were all significantly reduced following the inactivation of YgeK in APEC. Then, we performed transcriptome sequencing to analyze the functional pathways involved in the biological processes. Results suggested that ETT2 transcriptional regulator YgeK plays a crucial role in APEC virulence. These findings thus contribute to our understanding of the function of the ETT2 cluster, and clarify the pathogenic mechanism of APEC.

## 1. Introduction

Extraintestinal pathogenic *Escherichia coli* (ExPEC) is an important zoonotic pathogen that can cause serious extraintestinal infections in humans and animals [1]. ExPEC can be divided into avian pathogenic *E. coli* (APEC), uropathogenic *E. coli* (UPEC) and new meningitis *E. coli* (NMEC) according to the affected host and organs [2,3,4,5]. APEC is the leading cause of avian colibacillosis, and one of the major causes of morbidity, mortality, and economic loss in the global poultry industry [6]. Known virulence factors of APEC are adhesin, toxin, iron-acquisition factor, lipopolysaccharide, and invasion factors *ibeA*, *ibeB,* and *ibeC* [7,8,9]. Several studies showed that APEC and human ExPEC share some virulence genes, and suggest that APEC may act as a virulence gene reservoir for ExPEC, presenting a threat to public health [3,10,11]. However, the pathogenic mechanism of APEC is still unknown, and difficulties still exist in the prevention and treatment of avian colibacillosis. Therefore, it is necessary to study the pathogenic mechanism of APEC to promote to development of effective vaccines or look for new potential drug targets to control APEC.

Protein secretion systems play important roles in prokaryotes, including in the defense against biological and chemical agents, and facilitating disease [12]. The Type III secretion system (T3SS) is the most common secretion system, and it is used to subvert eukaryotic signaling pathways in Gram-negative bacteria, such as *Yersinia* species, *Salmonella* enteria serovars, *Shigella* species, and *Escherichia* species [13,14]. *E. coli* type III secretion system 2 (ETT2) was first identified in enterohaemorrhagic *E. coli* O157: H7. The prevalence of the ETT2 cluster in APEC was higher than that in human ExPEC (UPEC and NMEC) [15,16,17]. The intact ETT2 locus is 29.9 kb with 35 genes, including *yqe* (ecs3703–3706), *yge* (ecs3707–3712), *epr* (ecs3716–3719), *etr* (ecs3720), *epa* (ecs3721–3726), and *eiv* (ecs3727–3737) [18]; however, not all 35 genes exist in clinical *E. coli* isolates. Though the presence of ETT2 is often accompanied by extensive deletions and insertions in clinical *E. coli* isolates, some studies showed that the ETT2 cluster or the genes within the cluster were vital for virulence in ExPEC strains, such as the ETT2 cluster in UPEC, ETT2 ATPase EivC in APEC, and ETT2 transcriptional regulator YqeI in APEC [19,20,21].

YgeK, which encodes a transcription regulator, is located in the ETT2 cluster. It was identified as an important regulator of gene expression in EHEC [22]. However, the function of transcriptional regulator YgeK in APEC remains unknown. In this study, we evaluated the role of YgeK in a clinical APEC isolate with an intact ETT2 cluster. First, we carried out a BLAST search of the YgeK amino acid sequence in the clinical APEC isolate AE81, *E. coli* K12, and *E. coli* O157: H7. We then constructed ygeK mutant strain AE81Δ*ygeK* and determined the impact of inactivation of YgeK on APEC characteristics. We also analyzed the affected pathways by transcriptional regulator YgeK by transcriptome sequencing. We confirmed that transcription regulator YgeK played an important role in APEC, thus providing new clues for future research into the pathogenic mechanism of APEC.

## 2. Results

### 2.1. Amino Acid Sequence of YgeK in AE81 Differed from That in E. coli K12

**Figure 1:**
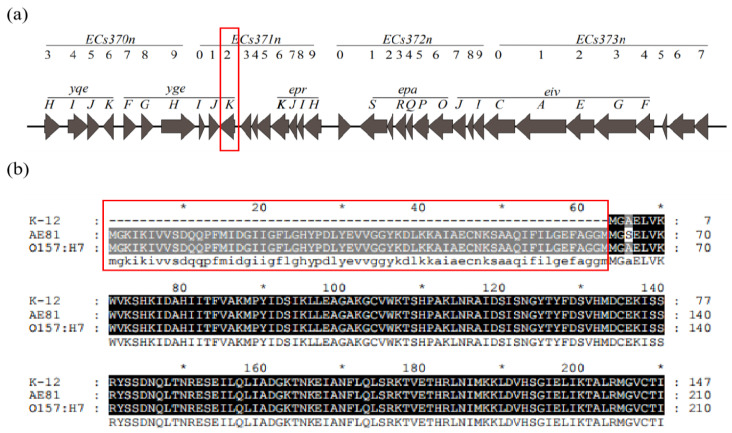
(**a**) ETT2 cluster containing 35 genes from ECs3703 to ECs3737 in AE81; (**b**) alignment analysis of YgeK amino acids in *E. coli* K12, AE81, and *E. coli*_O157: H7. Black filled area has the same amino acid, and gray area has different amino acids.

A schematic diagram of the ETT2 cluster is shown in Figure 1a, according to the AE81 sequence. Full-length YgeK in AE81 was composed by 210 amino acids. The amino acid sequence was the same in *E. coli* O157: H7 except for the 66th amino acid, which was serine (S) in AE81 and alanine (A) in *E. coli* O157: H7. However, the sequence in *E. coli* K12 was missing 189 bp (63 amino acids) at the 5′ end compared with the sequences in AE81 and *E. coli* O157: H7 (Figure 1b).

### 2.2. Inactivation of YgeK Influenced Morphological Structure and Motility of AE81

**Figure 2:**
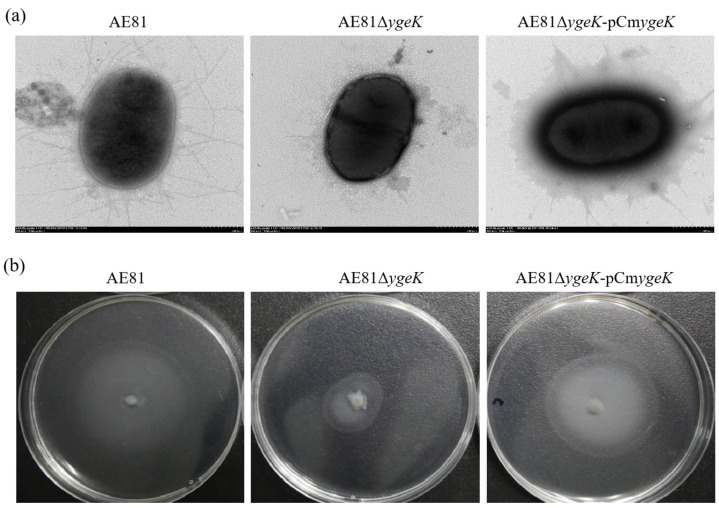
(**a**) Bacterial micromorphology of AE81, AE81Δ*ygeK*, and AE81AE81Δ*ygeK*-pCm*ygeK* observed by transmission electron microscopy (×10,000). (**b**) Motilities of AE81, AE81Δ*ygeK*, and AE81AE81Δ*ygeK*-pCm*ygeK* on semisolid medium.

Mutant strain AE81Δ*ygeK* and complemented strain AE81Δ*ygeK*-pCm*ygeK* were successfully constructed, and AE81Δ*ygeK* also showed a normal growth rate lysogeny broth (Figure S1). However, there were notable differences in morphological structure between AE81 and AE81Δ*ygeK*. Under transmission electron microscopy, there were long curved flagella covering the surface in AE81. In contrast, flagellar production was impaired in mutant strain AE81Δ*ygeK*, and only one flagellum was observed on the surface of AE81Δ*ygeK*. Flagellar formation was partially recovered in the complemented strain, and a few broken flagella appeared on the surface of AE81Δ*ygeK*-pCm*ygeK* (Figure 2a). In addition, after incubation on semisolid LB agar plates for 8 h at 37 °C, the swarming circle of AE81Δ*ygeK* was much smaller than that of AE81 and AE81Δ*ygeK*-pCm*ygeK*, but the wild-type phenotype was restored in AE81Δ*ygeK*-pCm*ygeK*, indicating that transcription regulator YgeK upregulates the motility of AE81 (Figure 2b).

### 2.3. YgeK Played an Important Role in AE81 Serum Resistance 

**Figure 3:**
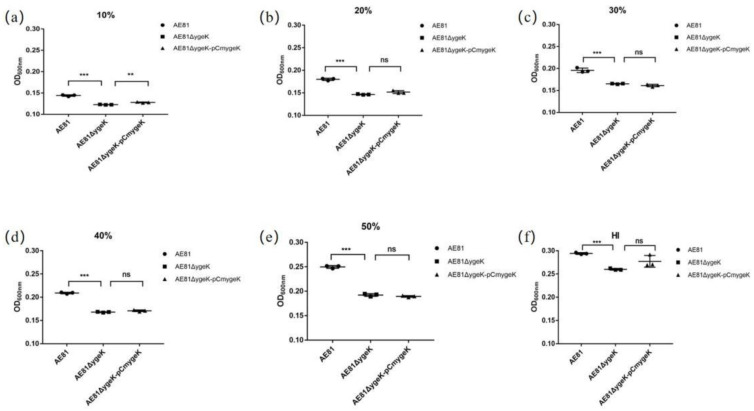
Bacterial resistance of AE81, AE81Δ*ygeK*, and AE81AE81Δ*ygeK*-pCm*ygeK* to SPF chicken serum. SPF chicken serum was added at concentrations of 10%, 20%, 30%, 40%, 50%, and heat-inactivated (HI). (**a**) SPF chicken serum was added at concentrations of 10%; (**b**) SPF chicken serum was added at concentrations of 20%; (**c**) SPF chicken serum was added at concentrations of 30%; (**d**) SPF chicken serum was added at concentrations of 40%; (**e**) SPF chicken serum was added at concentrations of 50%; (**f**) SPF chicken serum was heat-inactivated. Growth was determined using a BioTek Eon plate reader. Turbidity at 600 nm was measured each hour (** *p* < 0.01, *** *p* < 0.001, ns: no significance).

AE81, AE81Δ*ygeK*, and AE81Δ*ygeK*-pCm*ygeK* showed similar survival abilities in PBS and 10% SPF chicken serum. After incubation with 20%, 30%, 40%, and 50% serum, the survival capacity of AE81Δ*ygeK* was significantly lower than that of AE81 (** *p* < 0.01, *** *p* < 0.001). The bactericidal survival activity of AE81Δ*ygeK*-pCm*ygeK* was intermediate between that of AE81 and AE81Δ*ygeK*-pCm*ygeK* (Figure 3), showing that YgeK was crucial for serum resistance in AE81. Results of plasmid control are shown in Figure S2.

### 2.4. YgeK Upregulated APEC Capacity to Adhere to DF-1 Cells

**Figure 4:**
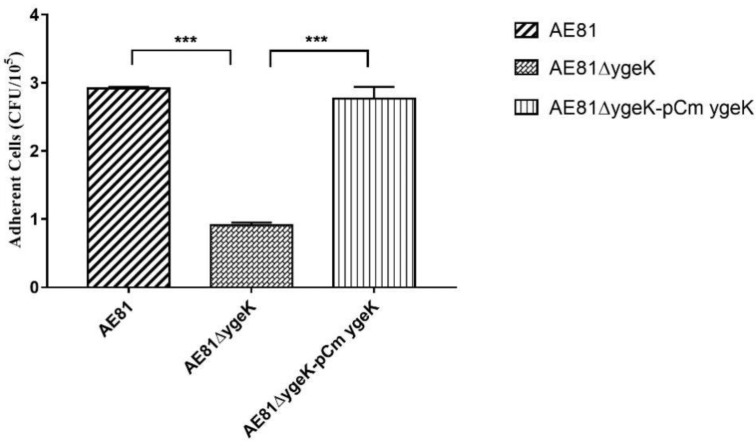
Bacterial adhesion assay with avian DF-1. Inactivation of YgeK decreased adhesion to avian DF-1 cells compared with wild-type AE81 and complemented AE81Δ*ygeK*-pCm*ygeK* strains. Values are the average of three independent experiments. Error bars indicate standard deviation. *** *p* < 0.01.

We investigated the role of YgeK in bacterial adhesion by infecting DF-1 chicken fibroblasts with AE81, AE81Δ*ygeK*, or AE81Δ*ygeK*-pCm*ygeK*. AE81Δ*ygeK* showed decreased adhesion to DF-1 cells compared with AE81 and AE81Δ*ygeK*-pCm*ygeK* (Figure 4). Results of plasmid control are shown in Figure S3. These results suggested that YgeK upregulated the capacity of APEC to adhere to DF-1 cells.

### 2.5. Transcriptional Profiling of AE81 and AE81ΔygeK

**Figure 5:**
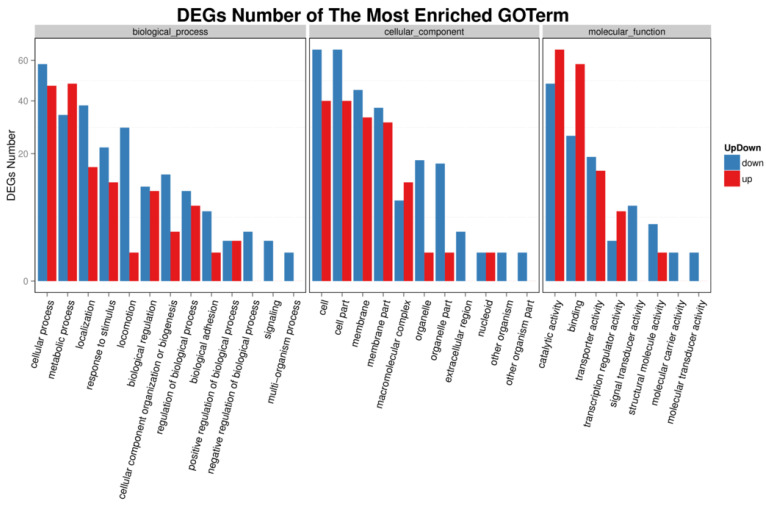
DEG GO classification analysis of wild-type AE81 and mutant AE81ΔygeK strains. DEGs were mainly enriched in biological process, cell composition, and molecular function. Downregulated genes indicated in blue bars; upregulated genes indicated in red bars.

**Figure 6:**
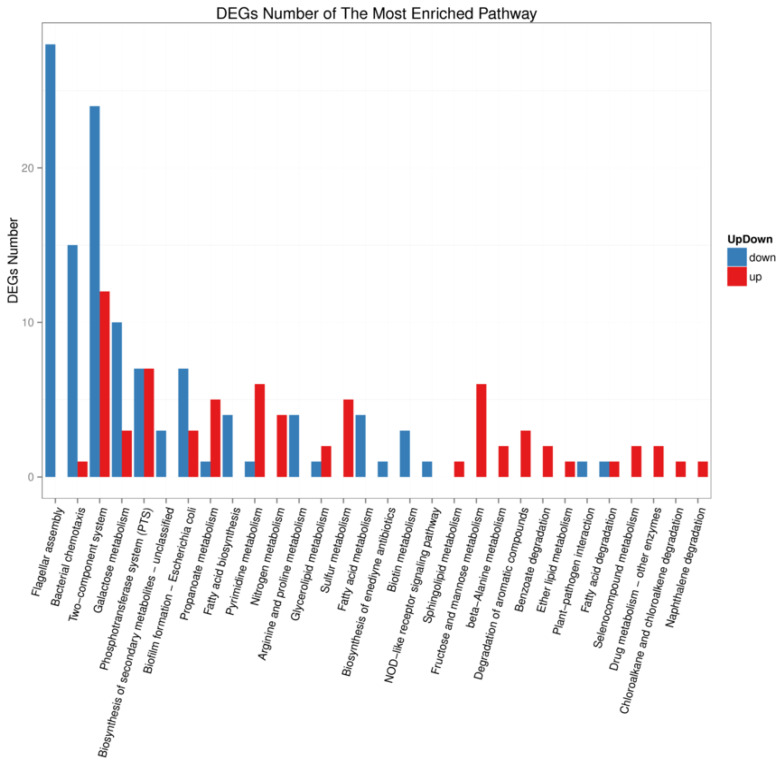
KEGG pathway classification analysis of wild-type AE81 and mutant AE81Δ*ygeK* strains. Downregulated genes indicated in blue, and upregulated genes in red.

We investigated the functional pathways affected by transcriptional regulator YgeK by transcriptome sequencing. A total of 4028 genes were quantified, and a total of 275 gene transcripts were defined as differentially expressed genes (DEGs) after deleting YgeK, including 137 upregulated and 138 downregulated DEGs (*p* ≤ 0.05, fold change ≥2.0) (Table S1). The most enriched Gene Ontology (GO) terms of DEGs were cellular process, metabolic process, and localization in biological process, cell, cell part and membrane in cellular component, catalytic activity, binding, and transporter activity in molecular function (Figure 5). The most enriched DEGs pathways included flagellar assembly, bacterial chemotaxis, two-component system, and biological adhesion (Figure 6).

### 2.6. YgeK Upregulated Flagellar and Virulence-Associated Genes

**Figure 7:**
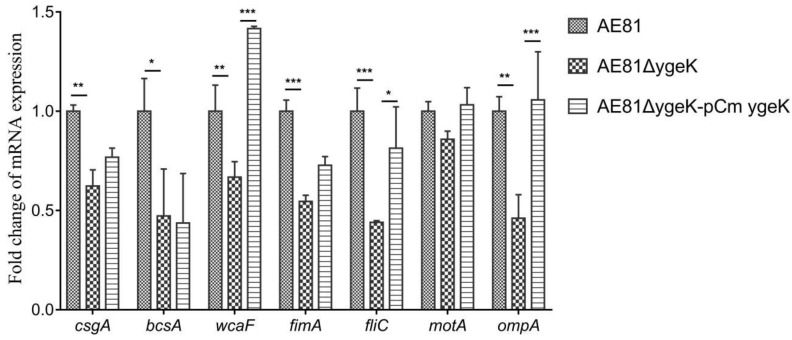
Comparative gene transcription levels (cDNA abundance). Transcript levels of biofilm genes *csgA*, *bcsA*, and *wcaF*, flagellar genes *fliC* and *motA*, fimbrial gene *fimA*, and virulence gene *ompA* in AE81, AE81Δ*ygeK*, and AE81Δ*ygeK*-pCm*ygeK* detected by real-time PCR. Results shown as expression ratios relative to expression in wild-type strain AE81. Error bars indicate standard deviation. Statistical significance assessed using two-way ANOVA (* *p* < 0.05; ** *p* < 0.01; *** *p* < 0.001).

We determined the transcript levels of biofilm-associated genes *csgA*, *bcsA*, and *wcaF*, flagellar genes *fliC* and *motA*, fimbriae gene *fimA*, and virulence gene *ompA* in AE81, AE81Δ*ygeK*, and AE81Δ*ygeK*-pCm*ygeK* by qRT-PCR. There were no changes in the transcript levels of *motA* in AE81Δ*ygeK*. The transcript levels of *csgA*, *bcsA*, *wcaF*, *fliC*, *fimA*, and *ompA* were significantly decreased in AE81Δ*ygeK* compared with AE81, and were restored in AE81Δ*ygeK*-pCm*ygeK* (Figure 7). These results confirmed that YgeK significantly upregulated the transcription of several flagellar and virulence-associated genes.

## 3. Discussion

Due to its presence in nonpathogenic *E. coli* K12 and pathogenic *E. coli*, whether ETT2 cluster can be used as a marker for detecting bacterial virulence is still inconclusive. However, *E. coli* K-12 did not possess an intact ETT2 cluster, and an intact ETT2 cluster or the genes encoded by the cluster affect the pathogenicity of ExPEC [19,20,23]. Transcriptional regulator YgeK is considered to be a pseudogene in *E. coli* K12, but it affects virulence in *E. coli* O157: H7. In this study, we analyzed the amino acid sequence of YgeK in different *E. coli* strains and found that the amino acid sequence of YgeK in AE81 was the same as that in *E. coli* O157: H7 except for one base difference, but both were significantly different from the sequence in *E. coli* K12. Each protein has a characteristic and unique amino acid sequence. The unique sequence or order of amino acids dictates the 3D conformation that the folded protein has, and this conformation determines the function of the protein. Thus, the similarity of the amino acid sequence of AE81 and *E. coli* O157: H7 provides the possibility of similar functions.

As expected, the inactivation of transcriptional regulator YgeK in AE81 led to a reduction in many bacterial functions, including flagellar formation, motility, and bactericidal activity, and adhesion ability. Flagella are more locomotive organelles in *E. coli*, and critical for biofilm formation and bacterial virulence [24,25]. Several studies revealed that transcriptional regulators YqeI and ATPase EivC both influence APEC’s flagella and motility [20,21], and this study supplements another transcriptional regulator in the ETT2 cluster that has an effect on APEC’s flagella and motility. Serum resistance is a prerequisite for septicemia, which is a typical symptom of avian colibacillosis [26]. The ETT2 cluster or the genes located at the ETT2 cluster are involved in serum survival of septicemia *E. coli*, NMEC, APEC and UPEC [19,20,27,28]. Deletion of YgeK attenuated serum resistance, supporting a vital role of the ETT2 cluster in serum resistance. In addition, adhesion to the host cells is an initial and important step in APEC pathogenesis. Meningitis-causing *E. coli* strain K1 with mutant ETT2 exhibited defects in invasion and intracellular survival compared with the parental strain. Yao et al. (2009) indicated that ETT2 was necessary for the pathogenic interaction between the *E. coli* K1 strain and host cells [28]. In this study, the inactivation of YgeK reduced the adhesion of APEC to DF-1 cells.

Since transcription regulator YgeK significantly influences APEC characteristics, we detected the effect of YgeK on the transcriptional level of other genes using transcriptome sequencing. Transcriptional analysis revealed that the top three most enriched pathways were flagellar assembly, bacterial chemotaxis, and two-component system. The expression of differently expressed flagellar genes was all down, which explained the differences in flagellar formation and motility between AE81 and AE81Δ*ygeK*. The motility and transcriptional level of flagellar genes in UPEC changed after deleting either the intact ETT2 or just part of this cluster [19]. 

A variety of transcription units in APEC have been reported, and an intricate regulatory network connecting various APEC virulence factors exists in APEC. These reported genes include global transcriptional regulators and two-component systems. They often affect APEC motility, biofilm formation, or other characteristics. For example, YjjQ, a global transcriptional regulator in APEC, affects APEC’s virulence through iron uptake [29]; McbR, acts as a transcriptional regulator involved in biofilm formation and the H_2_O_2_ stress response [30]; KdpDE, BarA-UvrY, and PhoPQ, as two-component systems in APEC, are all involved in APEC’s virulence [31,32,33]. There is a connection between different regulatory units. For example, phosphorylated CpxR directly regulates the expression of a Type VI secretion system (T6SS2) and promotes the pathogenicity of APEC by binding to the hcp2B promoter region of T6SS2 [34]. The functional description of transcriptional regulator YgeK contributes to the analysis of APEC pathogenicity.

## 4. Materials and Methods

### 4.1. Amino Acid Analysis of YgeK among Different E. coli Strains

The sequence of the ETT2 cluster and YgeK in AE81 was obtained from whole-genome sequencing [21]. The whole-genome sequencing library was prepared by Novogene Co., Ltd. Through the quality control of the Novogene pipeline, raw data weree filtered to obtain the clean data. SPAdes 3.10.2 and Velvet 1.2.10 software was used to splice the preprocessed sequence fragments and assemble them into a contig containing the information of the entire bacterial genome. The YgeK sequences of *E. coli* K12 and *E. coli* O157: H7 were downloaded from National Centre for Biotechnology Information (NCBI). Homology including the deleted sequence was calculated. The multiple sequence alignment among amino acid sequences was estimated with Molecular Evolutionary Genetics Analysis Version 7.0 (Mega 7.0).

### 4.2. Construction and Verification of the ygeK Mutant Strain

The mutant strain was constructed using the lambda Red recombinase system [35]. According to the sequence of YgeK in AE81, two long homology arm primers were designed. The chloramphenicol resistant fragment was amplified with the pKD3 plasmid as the template, gel-recovered, and transformed into AE81 cells with pKD46 plasmids using Gene Pulser Xcell with 200 Ω and 2.5 kV (Bio Rad, Hercules, CA, USA). Mutant strain AE81Δ*ygeK* was identified by PCR and sequencing. The pCP20 plasmid was used to cure pKD3, and the mutant strain was named AE81Δ*ygeK*. Strains and the plasmids are listed in Table 1, and primers are listed in Table S2.

### 4.3. Construction and Verification of Complemented Strain

The complemented fragments were amplified using the AE81 genome as a template with primers C-*Bam*HI-*ygeK*-f and C-*Hin*dIII-*ygeK*-r. The fragments and plasmid pSTV28 DNA were digested with *Bam*HI and *Hin*dIII, recovered by glue, and ligated with T4 ligase. Complemented strain AE81Δ*ygeK*-pCm*ygeK* was generated by cloning the target genes into plasmid pSTV28 and transforming the resulting plasmid into AE81Δ*ygeK* cells. Strains were grown in lysogeny broth (LB) medium (tryptone, 1.0 g; NaCl, 1.0 g; yeast extract, 0.5 g with 100 mL ultrapure water; when necessary, medium was supplemented with 1.5 g agar) with chloramphenicol (Cm, 30 µg/mL) at 37 °C with biochemical incubator (Boxun, Shanghai, China).

### 4.4. Bacterial Growth Curves

The overnight cultures of AE81, AE81Δ*ygeK,* and AE81Δ*ygeK*-pCm*ygeK* were diluted to an optical density at 600 nm (OD_600_) of approximately 0.03 in LB medium. Bacteria were cultured at 37 °C with 150 rpm and each hour to monitor optimal density using a UV/Vis spectrophotometer (DU730, Beckman Coulter, Miami, FL, USA).

### 4.5. Micromorphology Observation by Transmission Electron Microscopy

To observe the micromorphology of AE81, AE81Δ*ygeK,* and AE81Δ*ygeK*-pCm*ygeK*, they were stationary cultured overnight at 37 °C in LB and then washed with phosphate buffer saline (PBS) three times. The bacteria were placed on a 200 mesh formvar-coated copper microscopy grid, incubated at room temperature, stained with 2% aqueous uranyl acetate for 30 s, and dried. The micromorphology of AE81, AE81Δ*ygeK*, and AE81Δ*ygeK*-pCm*ygeK* was observed using transmission electron microscopy (HitachiHT-7700, Tokyo, Japan).

### 4.6. Motility Assay

The motility ability of AE81, AE81Δ*ygeK*, and AE81Δ*ygeK*-pCm*ygeK* was determined with semisolid medium (1.0 g tryptone, 1.0 g NaCl, 0.5 g yeast extract, 0.25 g agar with 100 mL ultrapure water). Stationary bacterial cultures were diluted 1:100 into a fresh LB medium and then cultured overnight. After culturing overnight, bacteria were washed with PBS three times and concentrated (OD_600_ = 2.0). The concentrated bacteria were dripped on a 0.25% semisolid medium and incubated for 8 h at 37 °C. Motility was assessed by examining the radius of the bacterial migration of the plate [21].

### 4.7. Serum Bactericidal Assay

Overnight cultures were diluted 1:25 into LB medium and cultured to the logarithmic phase. Bacteria were incubated with 10%, 20%, 30%, 40%, and 50% diluted specific-pathogen-free (SPF) chicken serum (Gibco, Grand Island, NY, USA) or heat-inactivated serum at 37 °C [19]. Growth was determined using FilterMax F3 Multi-Mode Microplate Reader (Molecular Devices, CA, USA), and optical density at 600 nm (OD_600_) was measured each hour.

### 4.8. Bacterial Adhesion Assay

Chicken embryo fibroblast DF-1 cell monolayers were washed with Dulbecco’s modified Eagle’s medium (DMEM) without fetal bovine serum, and infected with bacteria at a multiplicity of infection (MOI) of 100 for 2 h at 37 °C under 5% CO_2_. After washing with PBS, cells were lysed with 0.5% Triton X-100, and bacteria were counted by plating on LB agar plates. Assay was performed three times.

### 4.9. RNA Extraction and Library Preparation for Transcriptome Sequencing

Total RNA was extracted from AE81 and AE81Δ*ygeK*, and RNA was purified and fragmented. Total RNA was examined by a NanoDrop nd-2000 spectrophotometer and Agilent Bioanalyzer 2100 (Agilent Technologies, Santa Clara, CA, USA). Sequencing libraries were generated following the manufacturer’s recommendations. Library quality was assessed, and the constructed library was sequenced using the Illumina HiSeq sequencing platform.

### 4.10. Differential Expression Analysis

Reads mapped to each gene were counted using HTSeq. The reads per kilobase of exon model per million mapped reads (FPKM) of each gene were calculated on the basis of gene length and the number of mapped reads. Resulting *p* values were adjusted using the Benjamini and Hochberg approach for controlling the false discovery rate; a *p*-adj < 0.05 was set as the threshold for a significant difference. GO enrichment analysis of different expressed genes (DEGs) was implemented with the GO seq R package. The Kyoto Encyclopedia of Genes and Genomes (KEGG) pathway was analyzed in KEGG mapper [36,37]. Transcriptome sequencing data were deposited into the NCBI Gene Expression database with SRA accession number SRR9835574.

### 4.11. Quantitative Real-Time PCR (qRT-PCR)

The expression of several DEGs was investigated with StepOnePlus™ Real-Time PCR System (Thermo Fisher Scientific, Waltham, MA, USA). Total RNA was isolated from bacterial cultures using Total RNA Extractor (Trizol) (Sangon Biotech). Contaminated DNA was removed from the samples with RNase-free DNase I (TaKaRa, Dalian, China). cDNA synthesis was performed using the PrimeScript RT reagent kit (TaKaRa) according to the manufacturer’s protocol. qRT-PCR was performed using SYBR Premix Ex TaqTM (TaKaRa) with specific primers. Relative gene expression was normalized to the expression of housekeeping gene 16 S via the 2^−ΔΔCt^ method (where Ct = cycle threshold). The assay was repeated three times. Primers used in this assay are listed in Table S2.

### 4.12. Statistical Analysis

All data were analyzed using the statistical software SPSS (v19.0) using one-way ANOVA. A paired t-test was used for statistical comparisons between groups. The level of statistical significance was set at a *p* value of 0.05.

## 5. Conclusions

This study first proved that transcriptional regulator YgeK located at the ETT2 cluster variously and robustly impacts APEC characteristics, including flagellar formation ability, motility, serum sensitivity, and adhesion ability. YgeK significantly upregulated the transcription of several flagellar and virulence-associated genes. Potential pathways explained for phenotypic differences further clarify APEC’s pathogenicity mechanism and the search for new prevention strategies. We clarified the role of the transcriptional regulator YgeK in APEC, and elucidated its regulatory network using transcription sequencing. These results define the critical role of YgeK in APEC’s pathogenicity, providing reference for research on ETT2 cluster function, and guiding the search for new drug targets and vaccines.

## Figures and Tables

**Table 1:** Strains and plasmid used in this study.

Strains or Plasmid	Genotype or Description	Source
Strains		
AE81	APEC clinical strain, isolated from lung	Laboratory stock
AE81Δ*ygeK*	AE81 *ygeK* deletion mutant	This study
AE81Δ*ygeK*-pCm*ygeK*	AE81Δ*ygeK* with the plasmid pCm*ygeK*, Cmr ^1^	This study
Plasmid		
pCm*ygeK*	pSTV28 with *ygeK* gene, Cmr ^1^	This study

^1^ Cmr, chloramphenicol-resistant.

## Data Availability

Transcriptome sequencing data were deposited into the NCBI Gene Expression database with SRA accession number SRR9835574.

## Supplementary Materials

The following are available online at https://www.mdpi.com/article/10.3390/ani11113018/s1. Table S1: Gene Information, Table S2: Primers used in this study. Figure S1: normal growth rate lysogeny broth. Figure S2: Results of plasmid control. Figure S3: Results of plasmid control.
