# Supplementary material for: Transcription Regulator YgeK Affects the Virulence of Avian Pathogenic Escherichia coli
Source: Animals (Basel). 2021 Oct 20;11(11):3018. doi: 10.3390/ani11113018 (PMC8614350; doi:10.3390/ani11113018)
Supplement: Supplementary file 1 [file animals-11-03018-s001.zip › animals-1261338-table S2.pdf]

Table S2. Primers used in this study.

Primer Name	Primer Sequence (5' -3')
pKD46-f	GATACCGTCCGTTCTTTCCTT
pKD46-r	TGATGATACCGCTGCCTTACT
pKD3-ygeK-CM-f	TTGTAATATTTCTGACTCACGATTTGTAAGTTGATTATCAGAGGAATACCGTGTAGGCTGGAGCTGCTT
pKD3-ygeK-CM-r	GATGATGGGATAATTGGATTCCTCGGACATTATCCTGATTTATATGAGGTCATATGAATATCCTCCTTAGTTC
ygeK-out-f	GGATAGCATAGGGAAGAACAGAG
ygeK-out-r	AATCCTTCTTGAAACCACCG
ygeK-in-f	TTTCCATACGCATCCTTTC
ygeK-in-r	TAGCGGAGTGTAACAAATCTG
C-*Bam*H I-ygeK-f	CGCGGATCCGATGGGAAAAATTAAAATTGT
C-*Hin*d III-ygeK-r	CCCAAGATTTTATATAGTGCACACACCCA
Rt-*csgA*-f	AGCGCTCTGGCAGGTGTTGT
Rt-*csgA*-r	GCCACGTTGGGTCAGATCGA
Rt-*motA*- f	GGCAATAATGGCAAAGCGAT
Rt-*motA*-r	CAGCGAAAACATCCCCATCT
Rt-*fliC*-f	CCTGAACAACACCACTACCA
Rt-*fliC*-r	TGCTGGATAATCTGCGCTTT
Rt-*fimA*-f	TGCTGTCGGTTTTAACATTC
Rt-*fimA*-r	ACCAACGTTTGTTGCGCTAC
Rt-bcsA-f	GATGGTACAAATCTTCCGTC
Rt-bcsA-r	ATCTTGGAGTTGGTCAGGCT
Rt-wcaF-f	TCTCGGTGCCGAAAGGGTTC
Rt-wcaF-r	ATTGACGTCATCGCCGACCC
Rt-ompA-f	GGTTTCAGGGTTGCTTTGTTG
Rt-ompA-r	GGTGTTTCCTACCGTTTCG
Rt-16s-f	TTTGAGTTCCCGGCC
Rt-*16s*-r	CGGCCGCAAGGTTAA
