# Supplementary figures and images for: Transcription Regulator YgeK Affects the Virulence of Avian Pathogenic Escherichia coli
Source: Animals (Basel). 2021 Oct 20;11(11):3018. doi: 10.3390/ani11113018 (PMC8614350; doi:10.3390/ani11113018)

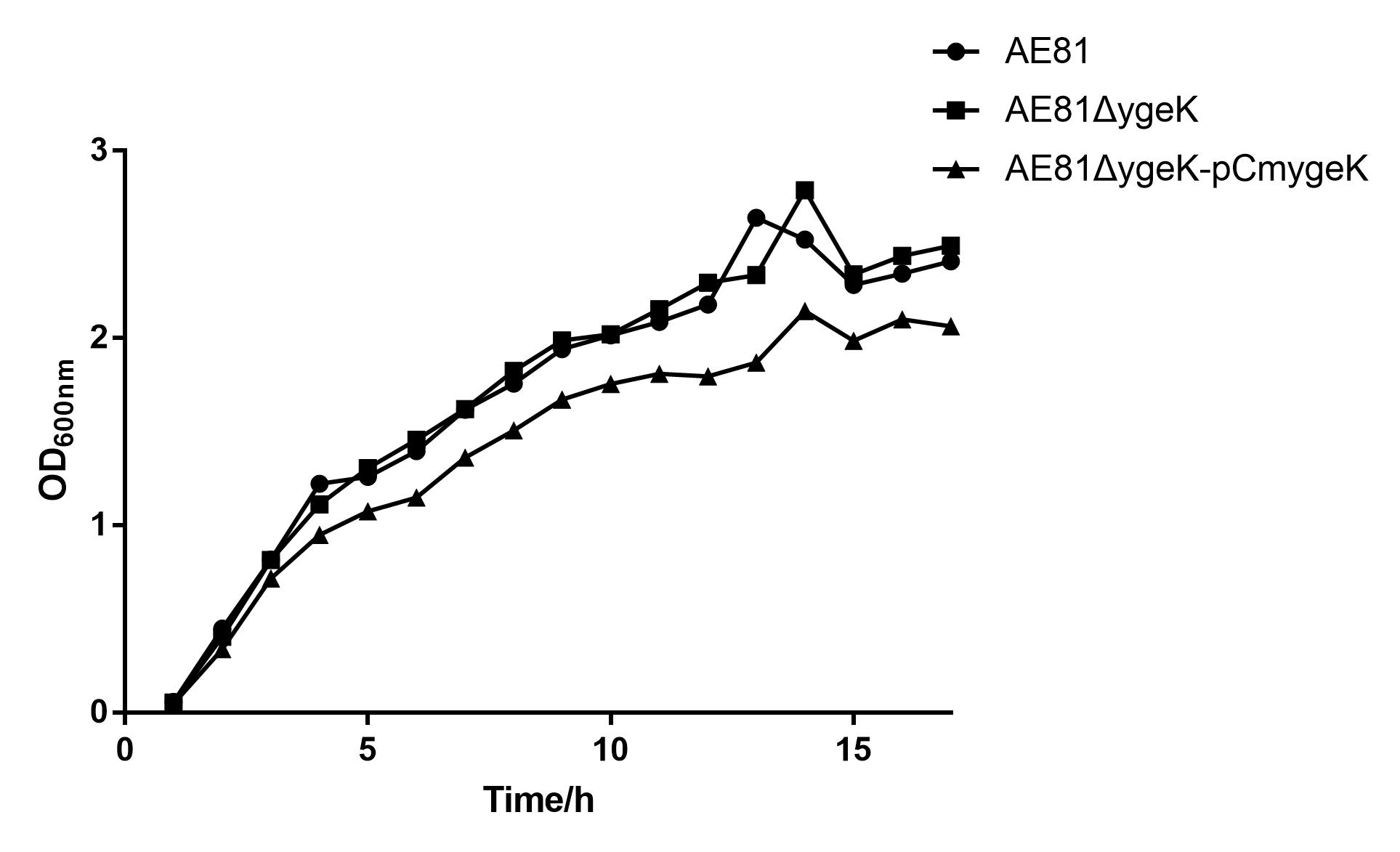

Supplement: Supplementary file 1 [file animals-11-03018-s001.zip › Figure S1.jpg]

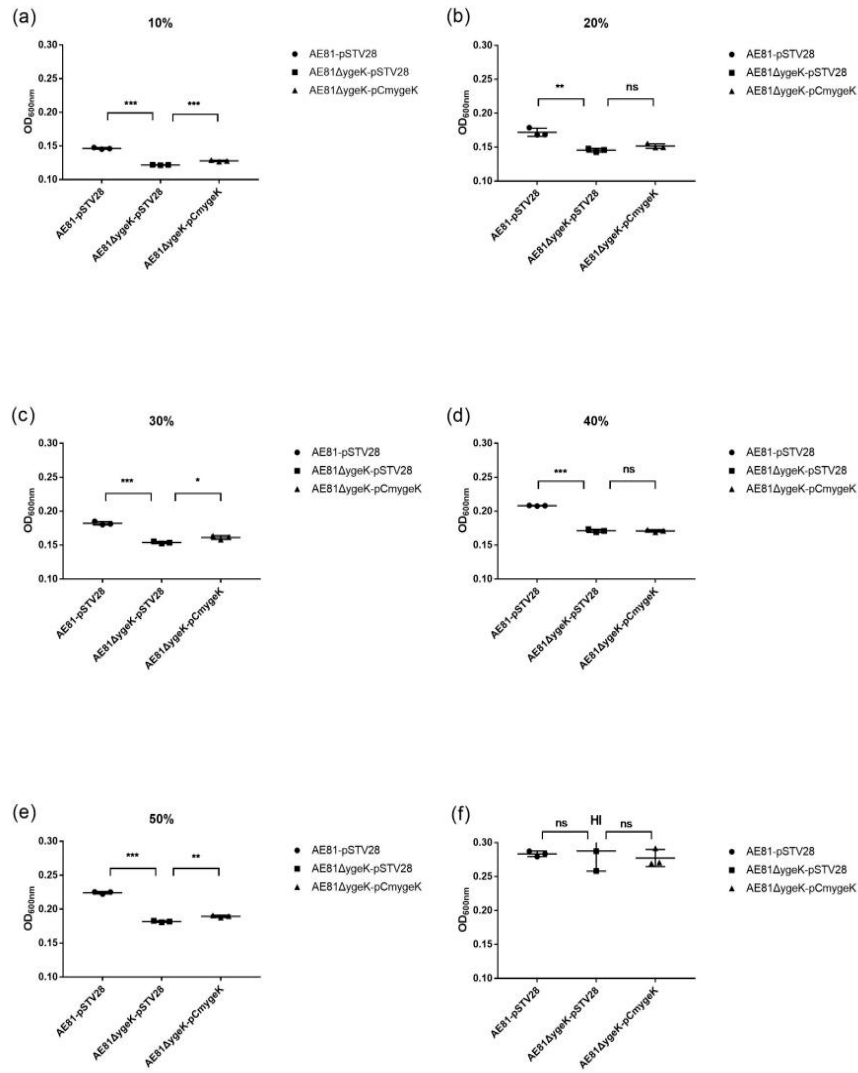

Figure S2. Bacterial resistance of AE81-pSTV28, AE81ΔygeK-pSTV28, and AE81ΔygeK-pCmycK to SPF chicken serum.

Supplement: Supplementary file 1 [file animals-11-03018-s001.zip › Figure S2.pdf]

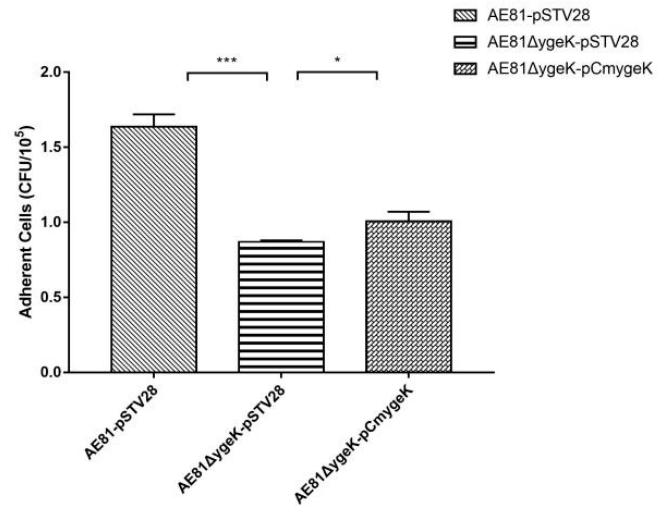

Figure S3. Bacterial adhesion assay with avian DF-1 of AE81-pSTV28, AE81ΔygeK-pSTV28 and AE81ΔygeK-pCmyeK.

Supplement: Supplementary file 1 [file animals-11-03018-s001.zip › Figure S3.pdf]
